# Do intentions for action penetrate visual experience?

**DOI:** 10.3389/fpsyg.2014.01265

**Published:** 2014-11-05

**Authors:** Robert E. Briscoe

**Affiliations:** Philosophy, Ohio UniversityAthens, OH, USA

**Keywords:** cognitive penetration of perception, two visual systems hypothesis, intention, consciousness, motor control

A now-famous study by Aglioti et al. (1995) involves a graspable version of the Ebbinghaus illusion (Figure 1). Aglioti and colleagues constructed a 3D version of the illusion, using thin solid disks. Subjects were asked to pick up the central disk on the left if the two central disks appeared identical in size, and to pick up the central disk on the right if they appeared different in size. The experimenters varied the relative sizes of the two target disks randomly so that in some trials physically different disks appeared perceptually identical in size, while in other trials physically identical disks appeared perceptually different in size. In selecting a disk in either trial condition, Milner and Goodale observe, “subjects indicated their susceptibility to the visual illusion” (1995/2006, p. 168): that is, their *choice* of which disk to pick up was determined by its apparent size rather than its real one. Nonetheless, the effect of the illusion was significantly less pronounced with respect to action, as measured by maximum grip aperture (MGA) in prehension, than with respect to conscious perceptual estimation (PE), as measured by the distance between thumb and forefinger in a manual estimate of disk size. Although the disk surrounded by small circles in the illusion display typically *looks* about 10% larger than the disk surrounded by large circles, the increase in MGA when reaching for the former disk exhibited a magnitude of around only 6%.

**Figure 1:**
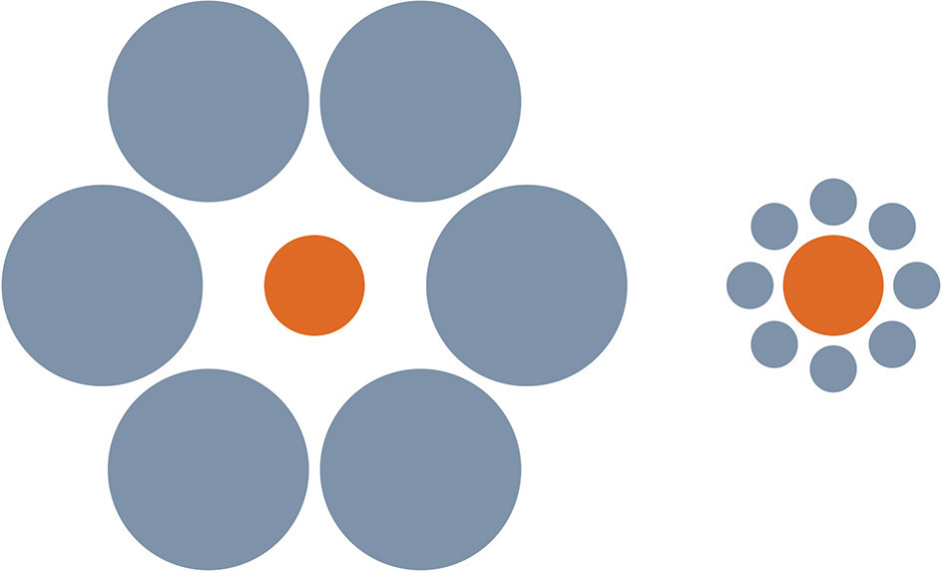
**The Ebbinghaus Illusion**. The disk on the left looks typically looks about 10% smaller than the disk on the right.

According to proponents of the dual systems model of visual processing (Milner and Goodale, 1995/2006), the illusion has a different effect on visual awareness than on visually guided grasping because the former makes use of different sources of visuospatial information than the latter. On this model, how the size of an object appears in conscious vision should not influence grip aperture, and, conversely, how the size of the object is represented by motor systems that guide grasping should not influence representation of its size in conscious vision.

At variance with this idea, however, Vishton et al. (2007) (experiment 3) found that the act of reaching for a disk in a 3D version of Ebbinghaus illusion significantly diminished the magnitude of the effect on subsequent PE for several minutes after reaching trials had ended (5.74% for PE vs. 6.10% for grasping). Strikingly, they also found (experiment 2) that when subjects were *merely informed* prior to engaging in PE trials that they would subsequently be required to grasp the disk that appeared larger, the effect of the illusion on PE was significantly diminished (6.18% for PE vs. 5.54% for grasping). “Simply listening to a description of a reaching task,” Vishton and co-authors write, “seems to affect size perception” (Vishton et al., 2007, p. 718).

These findings suggest that the phenomenal contents of visual experience can be cognitively penetrated: high-level information originating outside of the visual system seems to modulate the way an object's size visually appears. There are different possible mechanisms whereby such penetration might occur. Vishton and co-authors propose that “intending to reach for a target changes how the reacher perceives it” and that “action choice changes the nature of visual size perception” (p. 718). But how does action selection have this effect? One possibility (a) is that an abstract, high-level intention to act—either a “distal” or “proximal” intention in the sense of Pacherie (2008)—somehow exerts a direct influence on PE, say, by changing the relative weightings assigned by the visual system to sources of depth information such as binocular disparity, vergence, accommodation, and relative size. Since size estimation depends, in part, on perceived distance in depth, this could explain the influence of intention on perception. A second possibility (b) is that the relevant effect is brought about via lower-level motor representations that implement and provide kinematic and dynamical specification for the subject's high-level intention. This would arguably still count as a case of cognitive penetration if the lower-level, action-specifying motor representations carried information from the subject's high-level intention that influenced relative cue weighting or other visual computations. As Wu (2013) writes, “The key [to cognitive penetration of vision by intention] is not directness of link but (internal) informational transfer of an appropriate kind” (p. 662). A third possibility (c) looks to motor imagery elicited in the course of both experiments for the source of penetration. Possibility (c), however, is not entirely distinct from (a) and (b), since there is evidence that internally rehearsing the performance of an action activates representations at all levels in the motor processing hierarchy (for reviews, see Decety and Grèzes, 2006; Jeannerod, 2006). A final possibility (d) is that the effect is not due to motor representations at all, but rather to the subject's *beliefs* concerning the action that she has been requested to perform.^1^ Future studies will have to investigate which, if any, of these four explanations best accounts for the intriguing effects that Vishton and his co-authors have reported.

## Conflict of interest statement

The author declares that the research was conducted in the absence of any commercial or financial relationships that could be construed as a potential conflict of interest.

## Footnotes

^1^I am grateful to the reviewer for suggesting this possibility.
